# Needle-Knife Fistulotomy for the Rescue: An Unusual Cause of Iatrogenic Extrahepatic Biliary Obstruction

**DOI:** 10.1155/2018/8159451

**Published:** 2018-01-30

**Authors:** Laura L. Ulmer, Lokesh K. Jha, Neil Bhogal, Saurabh Kapur, Saurabh Chandan, Derrick Eichele, Ishfaq Bhat, Shailender Singh

**Affiliations:** Division of Gastroenterology and Hepatology, Department of Internal Medicine, University of Nebraska Medical Center, 982000 Nebraska Medical Ctr, Omaha, NE 68198-2000, USA

## Abstract

A 71-year-old male presented to our institution with cholestatic hepatitis after having recently undergone upper endoscopy for treatment of gastrointestinal bleeding. Further investigation with endoscopic retrograde cholangiopancreatography revealed a hemostatic clip on the ampulla of Vater. After initial attempts at cannulation of the common bile duct were unsuccessful, biliary decompression was achieved by use of needle-knife fistulotomy. A common bile duct stent was placed and the liver function tests improved prior to discharge.

## 1. Introduction

The most common causes of extrahepatic biliary obstruction in adults are choledocholithiasis, tumor compression, primary sclerosing cholangitis, parasitic infection, AIDS cholangiopathy, and malignant strictures [1]. Iatrogenic biliary obstruction is uncommon in the nontransplant setting and is typically associated with operative trauma during cholecystectomy [2]. Endoscopic clip placement is an established technique to achieve hemostasis in nonvariceal upper gastrointestinal hemorrhage. This technique is commonly used in the therapy for peptic ulcer disease, Dieulafoy lesions, Mallory-Weiss tears, and duodenal diverticular disease [3, 4]. Endoscopic clip placement is associated with low rates of rebleeding and typically has an excellent safety profile [5, 6].

## 2. Case Report

**Figure 1:**
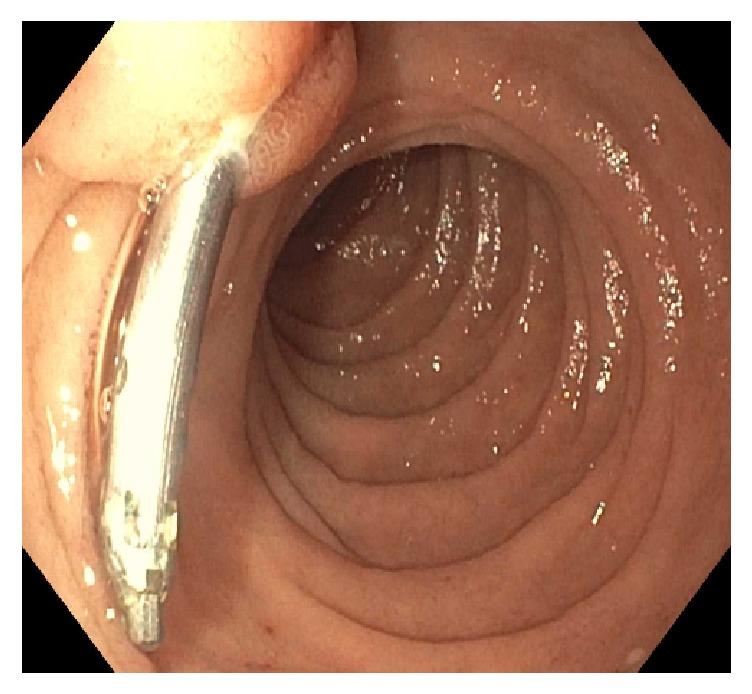
Hemostatic clip on the ampulla of Vater.

**Figure 2:**
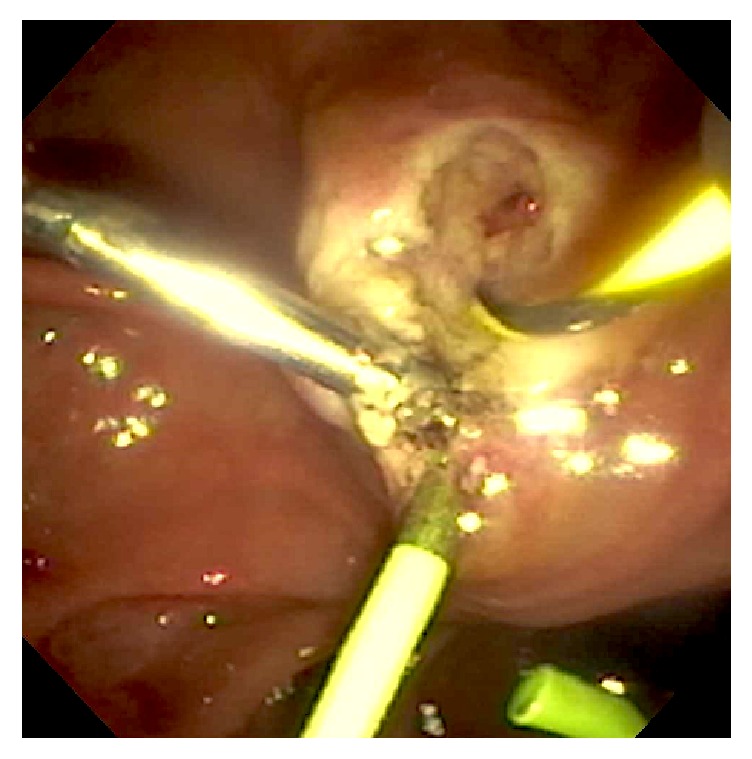
View of the ampulla of Vater following needle-knife fistulotomy, CBD cannulation, and pancreatic duct stent placement around the hemostatic clip.

A 71-year-old male with a history of hypertension and chronic kidney disease presented to an outside hospital with melena. An esophagogastroduodenoscopy (EGD) was performed and showed “a nipple-like bleeding vessel in the second portion of the duodenum,” which was treated with epinephrine and one hemostatic clip. The patient subsequently developed cholestatic hepatitis and was transferred to our center for further management. Labs upon admission revealed total bilirubin 6.1 mg/dL, alkaline phosphatase 219 U/L, aspartate aminotransferase 54 U/L, and alanine aminotransferase 59 U/L. An ultrasound of the abdomen showed multiple gallstones and a dilated common bile duct (CBD) measuring 1 cm. Subsequent endoscopic retrograde cholangiopancreatography (ERCP) showed a hemostatic clip on the ampulla of Vater, inadvertently placed at the time of recent EGD for bleeding control (Figure 1). Initially, the pancreatic duct was cannulated and injected. The pancreatogram was unremarkable and a 5 Fr × 3 cm pancreatic duct stent was placed. Wire-guided CBD cannulation was unsuccessful after a few attempts due to iatrogenic obstruction by the hemostatic clip. A needle-knife fistulotomy was then performed to obtain biliary access and the cholangiogram was unremarkable (Figure 2). A 10 Fr × 5 cm plastic CBD stent was placed to ensure CBD drainage. Liver function tests had improved by the following day and the patient was discharged home.

## 3. Discussion

Endoclip migration causing biliary obstruction has been previously described as a rare complication of cholecystectomy and is associated with choledocholithiasis and ascending cholangitis [7]. To our knowledge, this is the first described case of iatrogenic biliary obstruction secondary to inadvertent hemostatic clip placement during endoscopy. Biliary obstruction can lead to significant morbidity and mortality as it leads to biliary stasis and life-threatening ascending cholangitis [8]. ERCP is a safe and direct technique used for the evaluation and treatment of biliary disorders [9]. This case highlights the morbidity that can be associated with upper endoscopy as well as the importance of operator training in endoscopy, especially when interventions are performed near the ampulla. In situations where difficult biliary cannulation is encountered, needle-knife fistulotomy is a safe and effective tool when used by experienced endoscopists [10, 11].
